# Adolescent-Reported Interparental Conflict and Related Emotional–Behavioral Difficulties: The Mediating Role of Psychological Inflexibility

**DOI:** 10.3390/pediatric17020033

**Published:** 2025-03-11

**Authors:** Ludovica Giani, Cecilia Amico, Chiara Crepaldi, Marcella Caputi, Simona Scaini, Giovanni Michelini, Barbara Forresi

**Affiliations:** 1 Child & Youth Lab, Department of Psychology, Sigmund Freud University of Milan, Ripa di Porta Ticinese 77, 20143 Milano, Italy; amico.phd@milano-sfu.it (C.A.); chiaracrepaldi99@icloud.com (C.C.); s.scaini@milano-sfu.it (S.S.); g.michelini@milano-sfu.it (G.M.); b.forresi@milano-sfu.it (B.F.); 2 Dipartimento di Scienze della Vita, Università di Trieste, Via E. Weiss 2, 34128 Trieste, Italy; marcella.caputi@units.it; 3 Gruppo Studi Cognitivi, Cliniche Italiane di Psicoterapia Età Evolutiva, Corso San Gottardo 5, 20143 Milano, Italy

**Keywords:** interparental conflict, psychological inflexibility, emotional–behavioral difficulties, adolescents, mediation

## Abstract

Background/Objectives: Interparental conflict, with its multiple dimensions, represents a risk factor for youth mental health, triggering a series of cascading processes. Despite recent evidence highlighting that psychological inflexibility is a risk factor for adolescents’ psychopathology after stressful events, a limited number of studies have investigated its role in family conflicts. This study aims to investigate whether psychological inflexibility mediates the impact of conflict characteristics (intensity, frequency, and resolution) and threat appraisal of interparental conflict on the psychological difficulties of adolescent offspring. Methods: A sample of 195 adolescents aged between 15 and 19 years old completed the Children’s Perception of Interparental Conflict Scale, the Strengths and Difficulties Questionnaire, and the Avoidance and Fusion Questionnaire for Youth on Google Forms. Results: The findings revealed that adolescents living in a family environment characterized by high levels of perceived interparental conflicts exhibit a wide spectrum of psychological difficulties, either emotional or behavioral, partially mediated by their psychological inflexibility. However, when conflict between parents is interpreted as threatening, adolescents’ inflexibility appears to mediate the relationship between conflict and psychological difficulties. Conclusions: While future studies are needed to better understand this association, psychological flexibility might represent a relevant treatment target in adolescents exposed to interparental conflict.

## 1. Introduction

Interparental conflict has been extensively studied in the scientific literature, and its potential impact on youths’ maladjustment and psychopathology is widely recognized [1,2,3,4,5,6,7,8,9].

Children and adolescents exposed to interparental conflict appear to be at heightened risk for developing problematic psychosocial functioning, showing a vulnerability that can lead to internalizing and externalizing symptoms over time [9,10,11,12,13]. Evidence also suggests a concurrent influence of age and gender. Specifically, interparental conflict seems to have a greater impact on males during preschool and school years, whereas its effects are more pronounced in females during adolescence [14].

However, given that interparental conflict is an inevitable aspect of family life, and many youths exposed to high levels of parental discord do not develop psychopathology [15], identifying risk and protective factors, as well as mediating mechanisms associated with adjustment, has been a central focus of research in the psychological literature.

As a result of research conducted in this field, the construct of interparental conflict has evolved over time, reflecting an increasingly nuanced understanding of its characteristics, dimensions, and implications. Early studies focused on the problematic nature of specific overt behaviors, such as physical or verbal violence. Over time, however, the perspective shifted toward a dimensional framework that conceptualizes conflict as a continuum of severity, encompassing a range of manifestations [9]. Within this framework, multiple dimensions of parental conflict have been identified [10,16] leading to a significant distinction between constructive and destructive conflict [17]. While the former involves respectful, emotionally modulated discussion, aimed at resolution, the latter is defined by violent, aggressive, and hostile behaviors—either verbally or non-verbally expressed—that do not seek or promote resolution [18].

Moreover, other studies have shown that interparental conflict can trigger a series of processes impacting youth’ mental health [19], with two primary mechanisms identified as particularly relevant. The first highlights the mediating role of parenting and the parent–child relationship [13], while the second focuses on youths’ perceptions of marital conflict, which are shaped by contextual, developmental, cognitive, and emotional factors [18,20]. With regard to parenting, research suggests that the impact of interparental conflict occurs indirectly via the ’spillover’ of negative emotional and behavioral patterns from the couple’s relationship into the parent–child dynamic. This spillover, in turn, is associated with adverse psychological outcomes in offspring [9,21,22,23,24]. Conversely, according to Grych and Fincham’s [18] cognitive–contextual model, the effects of parental conflict on youths are shaped both by the characteristics of the conflict (“What is happening?”) and by the youths’ cognitive appraisal of the situation, through primary and secondary processes. Primary processing involves the child or adolescent’s perception of specific characteristics of interparental conflict, such as frequency, intensity, and potential resolution. Based on this perception, youths assess the level of threat posed by the conflict, considering its potential impact on themselves, their parents, or their parents’ relationship. When a conflict is perceived as nonthreatening, they are likely to divert their attention away from it, without engaging in further processing. Conversely, a more in-depth cognitive processing might be hindered or disrupted when initial processing evokes a direct sense of threat and negative emotions [18]. In the secondary processing, youths further evaluate the meaning of the conflict for their well-being, through appraisals focused on causal stability, self-blame, and coping efficacy [18,25,26]. Emotional responses triggered during primary processing are typically adjusted or regulated through these secondary processing mechanisms.

Despite evidence indicating that youths’ vulnerability to marital conflict may be exacerbated by mediating factors related to their cognitive and emotional appraisal (e.g., emotional insecurity, coping efficacy [27,28]), no prior research has explored the potential mediating role of psychological flexibility. This construct, along with its conceptually related behavioral repertoire characterized by psychological inflexibility, represents a core construct in Acceptance and Commitment Therapy (ACT) [29]. The Hexaflex Model of Psychological Flexibility defines both psychological flexibility and inflexibility as multifaced constructs comprising six distinct yet interconnected processes. Psychological inflexibility, as conceptualized within the ACT framework [30,31], is characterized by both experiential avoidance, which refers to attempts to suppress, control, or escape from unwanted internal experiences, and cognitive fusion, which reflects an overidentification with thoughts and emotions, leading to rigid behavioral patterns. While these two processes may seem contradictory—one involving avoidance and the other involving excessive entanglement—they are in fact complementary aspects of inflexibility. Empirical research has consistently shown that individuals with high psychological inflexibility may alternate between these two tendencies: they may avoid distressing emotions in some contexts while becoming overly entangled with their thoughts in others [31,32].

Research has consistently demonstrated the association between psychological inflexibility and both distress and maladaptive functioning [33,34], as well as its predictive role in mental health outcomes [32,35,36]. However, only a few studies have examined (in)flexibility in individuals who experienced stressful or adverse events, highlighting the detrimental impact of inflexible behaviors on an individual’s ability to cope effectively with such experiences. Some studies have focused on adult populations [37,38,39,40,41], while others on adolescents [42,43] and young adults [44,45,46]. Research involving youths and young adults has explored the mediating role of psychological inflexibility in conditions such as the COVID-19 pandemic [44], early life trauma [43,46], childhood psychological abuse [45], and parental rejection [42]. These studies suggest that risk conditions may contribute to adverse outcomes through patterns of behavior characterized by psychological rigidity. For instance, higher levels of Adverse Childhood Experiences (ACEs) may increase psychological inflexibility, reducing the ability to distance oneself from negative thoughts and increasing the avoidance of distressing thoughts and emotions. This, in turn, may heighten the likelihood of developing depression and anxiety [46]. The existing literature has primarily focused on examining the general construct of psychological inflexibility as a mediator between ACEs and psychopathological outcomes, with only a limited number of studies exploring the distinct dimensions of psychological inflexibility [46], or specific aspects (e.g., avoidance) [45]. In conclusion, despite some preliminary support for the role of psychological inflexibility in adverse situations, few studies involved samples of adolescents, and none investigated the role of inflexible patterns of behaviors in family contexts characterized by high levels of marital conflict. Nonetheless, the existing literature highlights how different dimensions of interparental conflict could contribute to behavioral patterns that are typical of psychological inflexibility. High-frequency conflicts may reinforce experiential avoidance—a core component of psychological inflexibility—by encouraging adolescents to disengage from distressing familial experiences. In line with avoidance-based models of anxiety and distress [31], when individuals perceive situations as uncontrollable or threatening, they may disengage from processing their emotions adaptively, adopting conflict avoidance which is a predictor of psychological difficulties [47]. However, it seems that high frequency alone is less predictive of negative psychological outcomes than a high intensity of the conflicts [48], which can create aversive learning experiences, reinforcing both avoidance behaviors and cognitive fusion with negative beliefs about relationships and emotional experiences (e.g., “conflict is dangerous” or “strong emotions should be suppressed”), in line with psychological inflexibility. Moreover, when adolescents interpret conflicts as threatening—either to their own well-being or to family stability—they are more likely to engage in experiential avoidance and psychological inflexibility as a means of self-protection [15]. Conversely, adolescents who witness parents engaging in effective resolution strategies (e.g., compromise and emotional validation) are less likely to develop rigid avoidance tendencies, mitigating the negative effects of conflict on psychological difficulties [47]. Given that chronic exposure to marital discord has been linked to emotional dysregulation, maladaptive coping, and long-term psychological difficulties in adolescents, understanding the role of psychological inflexibility in this context is critical. An exploration of this topic could be useful to clarify mechanisms that may contribute to persistent distress and to inform interventions aimed at fostering resilience in youth facing high-conflict family dynamics.

To address this gap, the present study aims to investigate whether psychological inflexibility plays a role in the pathogenic process linking interparental conflict to adolescents’ psychological vulnerability. Specifically, this research examines the mediating role of psychological inflexibility in the association between adolescents’ primary processing of interparental conflict—including appraisal of conflict characteristics (intensity, frequency, and resolution) and perceived level of threat—and their emotional and behavioral difficulties [49]. The better identification of mediating variables could inform intervention and prevention strategies in cases of destructive interparental conflict.

The first part of the mediation chain is supported by studies demonstrating how parental conflict and problematic parenting can foster the use of inflexible regulation strategies such as cognitive fusion and experiential avoidance [50,51,52]. Specifically, drawing from the Emotional Security Theory (EST) [20] and the Family Stress Model [53], exposure to high-conflict family environments can heighten emotional insecurity, disrupt self-regulation, and reinforce rigid cognitive and behavioral patterns as a means of coping with stress. Additionally, research on experiential avoidance [54] suggests that children exposed to chronic conflict may develop avoidance-based strategies (e.g., emotional suppression, and rumination) that limit their ability to adapt flexibly to stressors.

The second part of the mediation chain is supported by studies in which high levels of psychological inflexibility have been consistently associated with worse mental health outcomes in adolescents [55,56,57]. According to the Psychological Flexibility Model by [42], two core components of psychological inflexibility, including cognitive fusion and experiential avoidance, may exacerbate emotional distress, undermining autonomous self-regulation and having a negative impact on mental health and well-being. Although research with children and adolescents is less abundant, existing evidence shows that psychological inflexibility is associated with both externalizing and internalizing problems, particularly with symptoms of anxiety and mood disorders [56]. Specifically, the study by Peng and colleagues [57], involving 916 students aged 11 to 19, found that perception of high parental rejection was associated with increased psychological inflexibility, which in turn correlated with higher levels of depression and lower life satisfaction.

Based on studies investigating the mediating role of inflexibility in the association between adverse events and adolescents’ psychological difficulties, we hypothesize that inflexibility will mediate the impact of interparental conflict on emotional and behavioral difficulties in adolescents.

## 2. Materials and Methods

### 2.1. Participants and Procedures

The present study, employing a cross-sectional design, focused on data collected during the third time point (T2-May 2022) of a larger longitudinal study on adolescents’ psychological well-being during the pandemic and post-pandemic periods, which began in May 2021. The sample consists of 195 adolescents aged between 15 and 19 years old (17.30 ± 1.22 yo; f = 79.49%), with 45.10% living in Northern Italy, 28.20% in Central Italy, and 26.70% in Southern Italy. Participants were recruited online through snowball sampling and were invited to complete a battery of three questionnaires on Google Forms, which was advertised via social media. This approach facilitated broader outreach, allowing responses from individuals across various geographic regions and backgrounds. Not only did this strategy enhance the sample size, but it also increased the diversity of the participant pool, including adolescents from different family environments and varying levels of conflict. To minimize potential selection bias related to family dynamics, participants were informed that the study aimed to investigate the general well-being of adolescents and their families in the post-pandemic period, with a focus on psychological health, family relationships, and coping strategies, rather than explicitly emphasizing interparental conflict. The recruitment process began by identifying an initial group of participants, known as ‘seeds’, who met the study criteria. These individuals (high school students from northern and central Italy) were invited to participate and asked to refer others who met the same criteria and might be willing to take part. At the same time, the questionnaire link was disseminated via social media. This referral chain continued to expand over time, and the sampling process concluded when the data collection period, defined by the researchers, came to an end.

### 2.2. Ethical Statement

Ethics approval was received from the Ethics Committee of the Sigmund Freud University (Prot. Nr. PBZGDX3OAYFCW288612). Informed consent was obtained from each participant via an online form at the beginning of this study, following a thorough explanation of the theoretical rationale, main objectives, methodological procedures, and data management. In line with recent debates [36], appropriately framed adolescent samples can provide their own consent on specific topics, and parental consent is not required in these cases.

### 2.3. Instruments

Adolescents provided self-reported data on sociodemographic information, perception of interparental conflict, and their emotional and behavioral difficulties. 

The Children’s Perception of Interparental Conflict Scale (CPIC Scale) [58,59] is a cognitive–contextual model-based instrument measuring specific aspects of interparental conflict from the child’s perspective. This psychometric tool is designed to explore three main dimensions of interparental conflict, namely, parental conflict characteristics, children’s reactions to conflict, and degree of children’s involvement in the conflict. The CPIC Scale consists of 48 items clustered in 8 subscales. Parental conflict characteristics are investigated through the following subscales: Frequency, Intensity, Resolution, and Content. Children’s reactions to parental conflict are assessed through the subscales of Self-blame, Perceived threat, and Coping efficacy, whereas children’s involvement in parental conflict is measured via the Triangulation subscale. Item responses are provided on a three-point Likert scale, where 0 corresponds to “False”, and 2 corresponds to “True”. Thus, a higher score indicates a greater perception of interparental conflict from the child perspective. For the purposes of the present study, four subscales of the CPIC Scale were included in the assessment. The Frequency (6 items), Intensity (7 items), and Resolution subscales (6 reversed items) were administered to explore the overall characteristics of interparental conflict, while the Perceived threat subscale (6 items) was used to investigate children’s reactions to the conflict. Additionally, the construct “Conflict Properties”, which includes the 19 items from the Frequency, Intensity, and Resolution subscales, was used to assess children’s overall perception of parental conflict (CPIC Total). The CPIC Scale exhibits robust psychometric properties, as demonstrated by Fosco and Grych [25] and by Grych and colleagues [54], who reported a good Cronbach’s α coefficient of reliability (α = 0.87). Reliability has been confirmed as very good in the current study both for the total score (α = 0.94) and for the subscales: Frequency (α = 0.83), Intensity (α = 0.87), Resolution (α = 0.86), and Perceived threat (α = 0.84).

The Strengths and Difficulties Questionnaire (SDQ) [60] administered in its self-report version for adolescents was used to assess participants’ emotional and behavioral difficulties. The questionnaire comprises 25 items divided into 5 subscales: Emotional symptoms, Conduct problems, Hyperactivity/inattention, Peer relationship problems, and Prosocial behavior. Responses are rated on a three-point Likert scale, ranging from 0 (“Not true”) to 2 (“Certainly true”). Scores on the subscales, except for Prosocial behavior, are summed to calculate a total emotional and behavioral difficulties score, ranging from 0 to 40, with scores above the cut-off of 20 indicating a clinically significant condition. The psychometric properties of this instrument have been evaluated in previous studies, showing good reliability for the total difficulties scale in the original version (α = 0.82) and marginally acceptable reliability (α = 0.52) in the Italian version [60,61]. In the current research, the total difficulties scale demonstrated sufficient reliability (α = 0.68), while emotional difficulties (α = 0.43) and behavioral difficulties (α = 0.54) showed low psychometric reliability. Given these findings, we opted to use the SDQ Total score as the dependent variable in our study.

The Avoidance and Fusion Questionnaire for Youth (I-AFQ-Y) [62] is one of the most widely used instruments for assessing psychological inflexibility in adolescents. Developed as a unidimensional measure, it primarily captures cognitive fusion and experiential avoidance as intertwined processes [62]. Although the Hexaflex model suggests that psychological inflexibility comprises up to six dimensions, empirical research indicates that a single-factor structure provides a better fit, particularly in youth populations (e.g., [63,64]). Cognitive fusion and experiential avoidance, while theoretically distinct, tend to be highly correlated in adolescents. Rigid avoidance of distressing thoughts and emotions often reinforces fusion with these experiences, making them difficult to disentangle psychometrically [30]. Administered in its Italian version, the I-AFQ-Y assesses the level of psychological inflexibility arising from high levels of cognitive fusion and experiential avoidance. Examples of items assessing cognitive fusion include “The bad things about myself must be true”, while items such as “I stop doing things that are important to me whenever I feel bad” capture experiential avoidance. The short form of the I-AFQ-Y consists of 8 items, with responses evaluated on a 5-point Likert scale, where 0 corresponds to “Not at all true” and 4 corresponds to “Completely true”. Therefore, higher scores indicate greater levels of psychological inflexibility. The psychometric properties of this instrument have been evaluated in previous studies, demonstrating good reliability in the original version (α = 0.83) and sufficient reliability in the Italian version (α = 0.69) [62,65]. In the present study, the I-AFQ-Y showed good reliability (α = 0.84).

### 2.4. Data Analysis

Descriptive statistics were performed for all participants. The Shapiro–Wilk test was used to assess the normal distribution for all study variables. Correlations were carried out to examine the degree of linear associations between adolescents’ perceptions of interparental conflict, psychological inflexibility, and emotional and behavioral difficulties. Between-group comparisons were performed to rule out the influence of gender as a potential confounding variable on self-reported emotional and behavioral difficulties, levels of psychological inflexibility, and the four dimensions of interparental conflict. Finally, two explanatory indirect effect models were performed to test whether psychological inflexibility mediates the relationship between interparental conflict and adolescent behavioral and emotional difficulties. The first model utilized the CPIC total score as the predictor of interparental conflict, while the second model focused on the CPIC Perceived Threat subscale as the predictor.

## 3. Results

### 3.1. Preliminary Analysis

For the first analysis, Shapiro–Wilk tests were performed to assess the normal distribution of the CPIC total score (W = 0.95, *p* < 0.001), SDQ total score (W = 0.98, *p* < 0.001), and I-AFQ-Y total score (W = 0.98, *p* < 0.001). As the variables were not normally distributed, non-parametric tests were performed. Before conducting mediation analyses, a series of Mann–Whitney U tests were carried out to identify potential gender differences among all the study variables, and Spearman correlations were run to evaluate the strength of associations among them. The Mann–Whitney U test results showed that females were more likely than males to report higher levels of global interparental conflict (U = 2446.50, *p* = 0.04), and perceived threat in conflictual dynamics between parents (U = 2089.00, *p* = 0.001). Females also reported more frequent conflictual relationships between parents (U = 2396.00, *p* = 0.026), and a greater tendency of parents to resolve conflicts (U = 2267.00, *p* = 0.009). However, no gender differences were observed in the intensity dimension of the adolescent-reported interparental conflict (U = 2719.50, *p* = 23). Additionally, females exhibited higher levels of emotional and behavioral difficulties (U = 2322.00, *p* = 0.014) and psychological inflexibility compared to males (U = 2072.00, *p* = 0.001). The results of Spearman’s bivariate correlation are presented in Table 1.

### 3.2. Main Explanatory Indirect Effect Analyses: Mediation Models

Following our data analysis plan, two separate mediation models, described by Hayes [66] as model 4 of mediation, were tested. In the first model, the adolescent-reported overall perception of the interparental conflict, represented by the CPIC total score, was the independent variable (X), adolescent emotional and behavioral difficulties, represented by the SDQ total score, were the dependent variable (Y), and psychological inflexibility, represented by the AFQ total score, was included as the mediator (M). In the second model, the same mediation model was applied but the independent variable was replaced with the CPIC subscale represented by perceived threat in interparental conflict.

**Figure 1:**
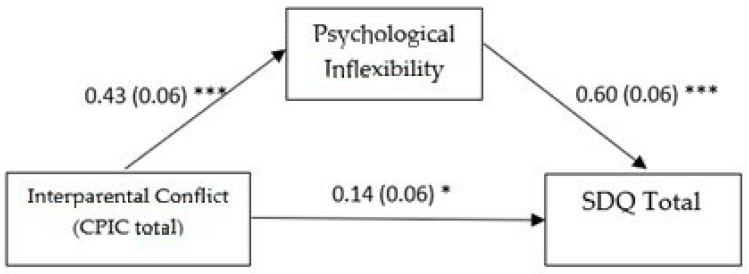
Mediation model with global adolescent-perceived interparental conflict as predictor. Note. Standardized coefficients are reported with standard errors in parentheses. Analyses were based on 5000 bootstrap samples with 95% bias-corrected confidence intervals; SDQ = strengths and difficulties questionnaire; * *p* ˂ 0.05; *** *p* ˂ 0.001.

In the first model (see Figure 1), all direct paths were significant. Notably, the adolescent-reported interparental conflict had a substantial effect on psychological inflexibility (ß = 0.428 SE = 0.065, *p* < 0.001, 95% CI [0.299, 0.556]). Both interparental conflict (ß = 0.145, SE = 0.0586, *p* = 0.0145, 95% CI [0.029, 0.260]) and psychological inflexibility (ß = 0.605, SE = 0.059, *p* < 0.001, 95% CI [0.489, 0.720]) significantly influenced adolescents’ overall emotional and behavioral difficulties. The total effect of the predictor and the mediator on the outcome was significant (ß = 0.404, SE = 0.066, *p* < 0.001, 95% CI [0.274, 0.533]), as was the indirect effect of the predictor on the outcome through the mediator (ß = 0.259, SE = 0.045, 95% CI [0.171, 0.350]). These findings might suggest that psychological inflexibility partially mediated the relationship between adolescents’ perception of interparental conflict and their psychological difficulties, with the indirect effect being stronger than the direct effect.

**Figure 2:**
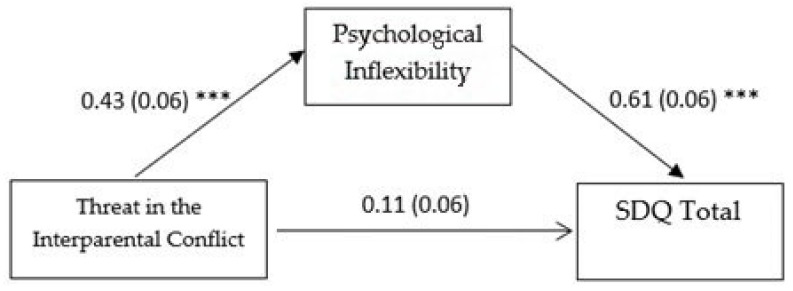
Mediation model with adolescent-perceived threat in the interparental conflict as predictor. Note. Standardized coefficients are reported with standard errors in parentheses. Analyses were based on 5000 bootstrap samples with 95% bias-corrected confidence intervals; SDQ = strengths and difficulties questionnaire; *** *p* ˂ 0.001.

In the second mediation model (see Figure 2), the direct effect of perceived threat in interparental conflict on psychological inflexibility (ß = 0.432, SE = 0.065, *p* < 0.001, 95% CI [0.304, 0.560]) was significant, as was the direct effect of psychological inflexibility on adolescents’ overall difficulties (ß = 0.618, SE = 0.059, *p* < 0.001, 95% CI [0.502, 0.735]). However, the direct effect of perceived threat in interparental conflict on adolescents’ overall psychological difficulties (ß = 0.112, SE = 0.059, *p* = 0.059, 95% CI [−0.005, 0.229]) was not significant. The total effect of the predictor and the mediator on the outcome was significant (ß = 0.379, SE = 0.067, *p* <0.001, 95% CI [0.248, 0.510]), as well as the indirect effect of the predictor on the outcome through the mediator (ß = 0.267, SE = 0.046, 95% CI [0.180, 0.363]). These results suggest that psychological inflexibility seem to play an important role in mediating the relationship between perceived threat in interparental conflict and adolescents’ emotional and behavioral difficulties.

## 4. Discussion

The present study aimed to explore psychological inflexibility as a potential mediator in the relationship between adolescents’ perception of interparental conflict—including intensity, frequency, resolution, and threat appraisal—and their psychological difficulties.

In line with previous research highlighting the detrimental effect of parental conflicts on psychological well-being of children and adolescents, our findings outline that destructive parental conflict seems to be linked to a higher risk of psychopathological development in these populations [9,10,11,12,67,68]. Furthermore, the current study contributes to the existing literature by highlighting the role of psychological inflexibility in mediating the impact of marital conflict on adolescents’ well-being.

Overall, the main finding of the present study highlights an association between living in a family environment characterized by destructive interparental conflicts—perceived not only as intense, frequent, and unresolved, but also threatening—and high levels of psychological inflexibility in adolescents which, in turn, may contribute to their psychological difficulties. This result aligns with previous studies suggesting that psychological inflexibility might act as a mediator between problematic parenting, particularly rejection and overcontrol, and psychological difficulties in adolescence [13,40,42,44].

Notably, inflexible patterns of behaviors appear to operate differently depending on whether the perception of the objective properties of conflict (i.e., intensity, frequency, and resolution) or threat appraisal is considered. In the mediation model where conflict properties were included as independent variables, psychological inflexibility showed a partial indirect effect between parental conflict and psychological outcomes. This might suggest that parental conflict with destructive properties (such as high intensity, frequency, and lack of resolution), may induce a state of activation or distress in adolescents, as thoroughly described by Cumming et al. [69]. Such distress could lead to cognitive fusion (e.g., regarding perceived responsibilities) or to rigid, avoidance-oriented behaviors (e.g., isolating oneself or excessive cell phone use) as strategies to protect the self, potentially jeopardizing adolescents’ mental health. A closer examination of these relationships seems to highlight that several interpretative pathways are plausible. For example, one possibility is that adolescents who experience higher levels of emotional and behavioral difficulties may tend to perceive the interparental relationship as more problematic, potentially due to heightened sensitivity or preexisting vulnerabilities. Alternatively, it is conceivable that elevated psychological inflexibility itself may predispose adolescents to greater emotional and behavioral challenges, independently of their perceptions of interparental dynamics. However, because this study employs a cross-sectional design, it is not possible to determine the direction of these effects definitively. Consequently, it remains unclear whether problematic perceptions of interparental conflict that arise from preexisting difficulties contribute to reduced psychological flexibility or simply coexist without a clear causal pathway.

Moreover, it is worth acknowledging that avoidance is not inherently maladaptive. In certain contexts, disengagement from stressors beyond one’s control—such as interparental conflict—can be a functional and adaptive coping strategy, particularly in adolescence. Research on coping mechanisms [70,71] supports the notion that situational avoidance or distraction can serve as protective responses when individuals have limited agency over stress-inducing circumstances.

In contrast, psychological inflexibility, as conceptualized in the Acceptance and Commitment Therapy (ACT) framework [31], differs from situational avoidance in that it reflects a rigid pattern of responding. Psychological inflexibility occurs when avoidance becomes pervasive, generalized, and motivated by an unwillingness to experience distressing emotions or thoughts, rather than by an adaptive appraisal of the situation. If avoidance extends beyond situational disengagement to a broader suppression of emotional processing—such as consistently avoiding discussions about emotions or interpersonal challenges—it may contribute to maladaptive outcomes, including increased psychological distress [32].

However, as the mediation effect is partial—and should be interpreted with caution due to the minimal difference between the direct effects in the two models—it appears that part of the impact of interparental conflict on adolescents’ symptoms may occur independently of inflexible behaviors, consistent with previous studies on problematic parenting [42]. This also aligns with prior studies on adverse or traumatic events, where the dose–response model suggests that psychological difficulties stem from a combination of exposure to the event (with its factual components, such as whether it is a one-time or repeated occurrence) and the perception of the event as stressful and dangerous [72,73,74,75]. According to this model, the risk of developing a psychological disorder increases in proportion to the intensity of the stressor [76,77], rather than solely being driven by subjective perceptions such as personal meanings or emotional activation, which many authors argue are more relevant than the actual danger associated with the event [78,79,80]. Some studies [26,68,81,82,83] consistently showed that specific characteristics of the conflict (e.g., duration, severity, and proximity) were directly associated with the psychological outcome.

Another possible explanation is that other factors might intervene in this relationship, such as parent–child triangulation [82], parent–child communication and self-esteem [83], as well as parental support, hostility, or intrusiveness [68].

Our findings suggest that when interparental conflict is perceived as threatening, psychological inflexibility is strongly associated with the relationship between conflict exposure and psychological difficulties. This could indicate that when adolescents interpret conflict as a direct threat, their ability to respond flexibly may be compromised, potentially leading to greater emotional and behavioral difficulties. In contrast, when considering the overall objective characteristics of interparental conflict—such as intensity and frequency—psychological inflexibility seems to be slightly less associated with its connection to psychological difficulties. This might suggest that while structural aspects of conflict contribute to maladjustment, other factors may also play a role in shaping adolescents’ responses.

Although the differences between these models may seem subtle, the statistical significance of the first model underscores the distinct pathways through which conflict characteristics may shape psychological adjustment. Given the high intercorrelations among CPIC subscales, it is essential to interpret these findings with caution, considering the overlapping yet conceptually distinct dimensions of interparental conflict.

Conversely, the second mediation model seems to emphasize a stronger mediating role of psychological inflexibility in the relationship between perceived threat and psychological difficulties. This suggests that when a conflict between parents is perceived as threatening and associated with danger, the ability to respond flexibly becomes crucial. Therefore, interpreting interparental conflict as threatening could impact on adolescents’ ability to react and cope flexibly in adverse situations, thereby increasing the probability of emotional and behavioral difficulties’ onset. This finding raises the hypothesis that growing up in an environment where interparental conflict is destructive may hinder the development of a flexible behavioral repertoire in children and adolescents [70,84]. This would be consistent with previous studies highlighting the detrimental effects of perceived parental conflict on the development of adaptive skills and socio-emotional growth in offspring [9,10,11,12,13].

However, the inability to use flexible strategies might also be interpreted as context-dependent, rather than a persistent pattern across all situations. In this case, when a conflict is perceived as threatening—whether for the adolescents themselves, their parents, or their parents’ relationship [18]—and negative emotions are triggered, the ability to engage in flexible behaviors might become impaired.

### Limits and Future Perspectives

When interpreting the implications of the current findings, it is important to recognize that the relationships among the variables are likely more complex than the analyses suggest, for several reasons. First, the present study relied on adolescents’ subjective estimates for “objective conflict characteristics” (intensity, frequency, and resolution). While an objective measure of event duration and intensity and resolution of conflict would be ideal, it may not always be practical. Additionally, the use of four subscales of the CPIC did not capture the distinction between primary and secondary cognitive appraisals of interparental conflict. However, the focus of this study on primary processing was driven by ecological considerations, aiming to minimize participant dropout and missing data due to an overly lengthy questionnaire battery.

Furthermore, while the correlation between adverse events and negative outcomes is widely accepted, the direction of the causality remains a topic of debate. It is now understood that not only children and adolescents exposed to stressful and adverse events may have higher polygenic score for psychiatric disorders [85], but also that certain psychological difficulties may influence the occurrence of specific family adverse events: as an example, ADHD may predict maltreatment from parents in adolescents [85]. Additionally, mental health may impact the perception and the recollection of adverse events. In the present study, ratings of conflict characteristics and perceived threat may have influenced each other, while also being influenced by symptom severity. Similarly, symptom severity might affect the use of flexible strategies in response to parental conflict.

Another limitation is the relatively small sample size, which reduces the statistical power of the analyses and limits the generalizability of the findings. The generalizability is further constrained by the online recruitment method, which excluded adolescents without Internet access, as well as the use of a convenience sample with a gender imbalance. Future research should further investigate the role of offspring gender in the context of parental conflict.

Moreover, data collection relied solely on self-reports, which may have introduced personal biases and confounding factors related to fears, prejudices, or even the adolescents’ emotional state at the time of responding. Future studies could assess psychological inflexibility using instruments other than the I-AFQ-Y. While the I-AFQ-Y conceptualizes psychological inflexibility as a single dimension primarily focusing on cognitive fusion and experiential avoidance, other processes involved in psychological inflexibility, as outlined in the Hexaflex Model (e.g., attachment to the conceptualized self, failure to act in accordance with core values), warrant specific investigation. Future research should also explore which aspect of psychological inflexibility—such as cognitive fusion, experiential avoidance, or other core processes from ACT—most strongly influences psychological difficulties.

Although this study offers valuable insight into adolescents’ perceptions of interparental conflict, it lacks the parents’ perspective. Future research could expand by including parents’ views on their conflictual situations and their beliefs about how these conflicts affect their children’s psychological well-being.

The cross-sectional design of this study presents a limitation that could be addressed in future longitudinal studies. Specifically, future research should explore the role of psychological inflexibility in adolescents exposed to parental conflict, distinguishing between internalizing and externalizing outcomes. The bidirectionality of this relationship could also be examined: while this study hypothesized that conflict and psychological inflexibility predict psychological difficulties, it is equally plausible that internalizing and externalizing difficulties increase adolescents’ rigidity in functioning, which could, in turn, influence the level of conflict between parents.

Finally, future studies could explore additional factors that help explain the relationship between interparental conflict and adolescent psychological well-being. Researchers should be aware that such factors and potential mediators may be influenced by contextual conditions, such as the family system (e.g., the impact of conflict on parenting as demonstrated by [68]) or the social context (e.g., the increase in parental conflict observed during the COVID-19 pandemic due to extended periods of confinement).

Despite these limitations, the relevance of the current findings remains significant. Our study suggests that psychological inflexibility, a core component of Acceptance and Commitment Therapy [29], may be a promising factor in addressing the psychological consequences of adolescents’ exposure to severe interparental conflict, including highly contentious separations or domestic violence. ACT-based interventions could be used to enhance psychological flexibility in adolescents, promote mental health [42], and potentially reduce the onset of emotional and behavioral difficulties [86,87,88].

## Figures and Tables

**Table 1:** Spearman correlations among the study variables.

Variables	2	3	4	5
1. CPIC Perceived threat	0.66 ***	0.39 ***	0.42 ***	−0.05
2. CPIC Tot	-	0.41 ***	0.42 ***	−0.01
3. SDQ Tot		-	0.69 ***	0.02
4. I-AFQ-Y Tot			-	−0.08
5. Age				-

Notes: *** *p* < 0.001. CPIC: children’s perception of interparental conflict scale; SDQ: strength and difficulties questionnaire; I-AFQ-Y: Italian version of the avoidance and fusion questionnaire for youth.

## Data Availability

Dataset available on request from the authors.
